# Dr. Edward F. Ashley

**Published:** 1911-04

**Authors:** 


Dr. Edward F. Ashley, bacteriologist of the quarantine hospital, New York harbor, died March 22, 1911, of cerebro-spinal meningitis, contracted while participating in the autopsies on the bodies of Greek steerage passengers brought to the port of New York, about a week ago, aged 30 years.

Dr. Ashley was a graduate of Yale and of the College of Physicians and Surgeons of New York.

